# Restarting Thalidomide-Dexamethasone Regimen in a Post-Abortive Female with Multiple Myeloma: Effective Clinical Response Possible

**Published:** 2017-10-01

**Authors:** Suhailur Rehman, S. H. Arif, Amit Kumar, A. Q. Khan

**Affiliations:** 1 Department of Pathology, Jawaharlal Nehru Medical College, Aligarh Muslim University, Aligarh, Uttar Pradesh, India; 2 Hind Institute of Medical Sciences, Lucknow, Uttar Pradesh, India; 3 Department of Orthopaedic Surgery, Jawaharlal Nehru Medical College, Aligarh Muslim University, Aligarh, Uttar Pradesh, India

**Keywords:** Multiple myeloma, Post-aborted woman, Thalidomide-dexamethasone therapy, High β-hCG levels

## Abstract

Nowadays, the prevalence of Multiple Myeloma (MM) seems to have been increasing among young females. Here, we report that thalidomide is contraindicated in pregnant women diagnosed with MM and those desirous of subsequent pregnancy. In this case report, we compared the clinical response of Thalidomide-Dexamethasone therapy in a post-abortive woman with persistently elevated β-hCG levels due to retained products of conception, undergoing hysterectomy later. This case report underlines the clinical significance of age, the effect of Thalidomide-Dexamethasone therapy even after initial discontinuation and the response to high β-hCG levels.

## Introduction

 Multiple myeloma is a neoplasm affecting bone marrow due to uncontrolled growth of plasma cells. The median age for diagnosis of multiple myeloma is 70 years and has a male predilection. Multiple myeloma in a woman of childbearing age is uncommon and the disease has a rapid downhill course^1^.

 Thalidomide dexamethasone therapy has proven useful and effective in the treatment of advanced and refractory myeloma, both as a primary and second-line treatment, with a high response rate and acceptable survival^2^. The main related complications are cytopenias, deep vein thrombosis, dry skin, constipation and paresthesia which require close monitoring^2^^.^^3^^,^^4^. Pregnancy is an absolute contraindication due to its teratogenic effects^5^.

Here, we present a case of a 32-year-old post- abortive female who was started on Thalidomide- Dexamethasone therapy, but the regimen had to be discontinued due to retained products of conception. The effective response was still possible by restarting the treatment with same drugs after the patient underwent hysterectomy as an absolute method of contraception. 

## Case report

A 32-year-old female presented to the gynecology department in JNMCH, AMU, Aligarh, India with eight weeks amenorrhoea, generalized body ache and painful gait. The patient was G4P3+0 L3, apparently normal, except for the pain in the last one-year for which she took medications as prescribed in tablet forms. There was no history of trauma or previous fracture/s.

A β HCG test confirmed pregnancy; per vaginum, per speculum and per abdominal examinations were within normal limits, the pregnancy being of 8 weeks. An orthopaedic reference raised the suspicion of multiple myeloma which was subsequently confirmed with radiological, haematological and urine examinations. The findings were as follows: 

 X-Ray Dorsolumbar Spine AP & Lateral View: showed multiple ill-defined lytic lesions with no definitive sclerosis. There was decreased height of vertebral body suggestive of collapse from T8, T11 and L1 to L4. 

X-Ray Skull Lateral view: showed multiple variable sized, ill-defined lytic areas involving the calvarium. 

**Fig 1:**
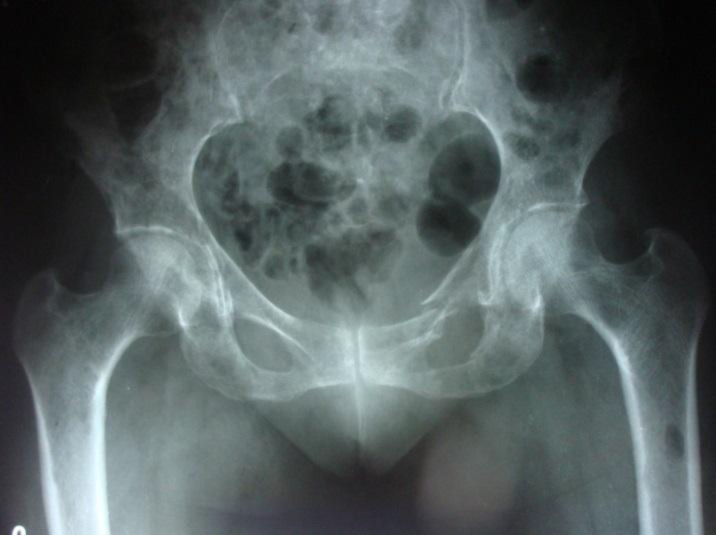
X-Ray Pelvis with both hips AP view- multiple lytic lesions in left subtrochanteric region.

 X-Ray Pelvis with both hips AP view: showed multiple lytic lesions in left subtrochanteric region (Figure 1).

**Fig 2:**
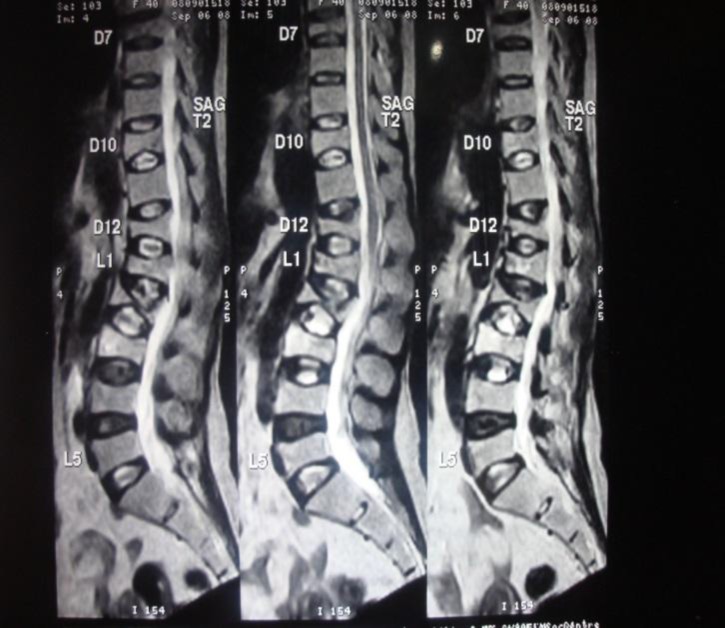
MRI- decreased height of vertebral body suggestive of collapse of T7, 10, 12 and L1-3.

MRI: decreased height of vertebral body suggestive of collapse of T7, 10, 12 and L1-3 (Figure 2).

Hematological investigation revealed increased E.S.R. and rouleaux formation with microcytic hypochromic anemia on peripheral blood smear. 

Biochemical investigations showed an increased S. Calcium concentration (17.4mg/dl). Baseline liver function test was within normal limit. 

**Fig 3:**
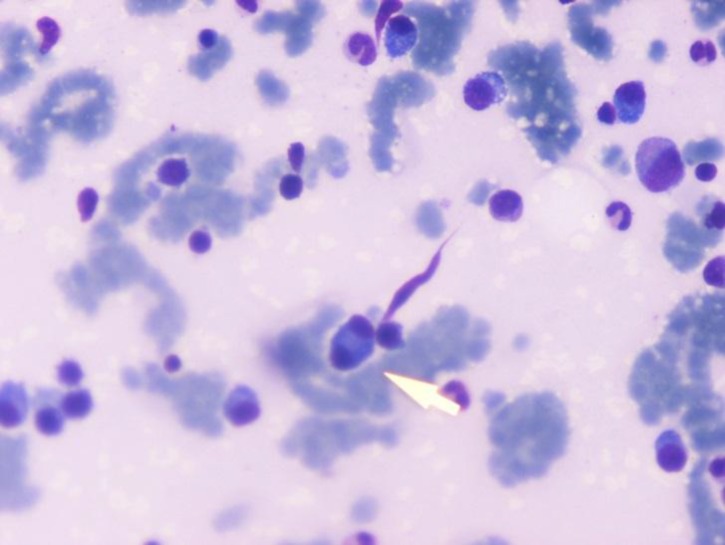
Leishman Stain bone marrow showing plasma cells with multinucleated plasma cell (100X)

Bone Marrow aspiration was performed and smear examination showed an increased number of plasma cells approximately 42%, including some abnormal cells and binucleate forms along with reversal of M: E ratio (Figure 3).

Bence-Jones proteinuria was seen in urine analysis. 

Serum protein electrophoresis showed Myeloma band in Gamma region with an M- spike of 4.54 gm/dl and serum β_2_-M microglobulin level of 3.2 mg/dl.

A diagnosis of multiple myeloma was made in the in an 8-week pregnant woman.

Medical termination of pregnancy was done using vacuum evacuation.

Total contraception was advised, and Thalidomide- Dexamethasone therapy (thalidomide 200mg orally in 24 hours and dexamethasone 50 mg orally for 4 days a week) started. Regular monitoring at weekly intervals was done using clinical sign and symptoms, urine examination, liver function tests and serum calcium. Bone marrow examination and serum protein electrophoresis were ordered at one-month interval.

The patient’s condition did not deteriorate, but there was no significant decline in levels of serum calcium, %age of plasma cells and the myeloma spike. Moreover, she developed nausea, vomiting and bleeding per vaginum. Clinical and radiological examinations did not reveal any new abnormality. A quantitative evaluation of hormonal profile was ordered, showing a progesterone level of 6.9 ng/ml, estrogen 25 ng/ml and β HCG 12 mIU/ml. A suspicion of pregnancy or associated neoplastic complications was aroused; the treatment was stopped and the patient thoroughly investigated with endometrial biopsy, but no obvious cause was found. 

The patient was convinced to undergo a total hysterectomy. Histopathological examination revealed retained products of conception, showing degenerating villi and hemmorrhage. After two weeks, β-hCG was not detectable in serum or urine, and other hormone levels were within normal limits. The patient was monitored for two months and treated conservatively. Thalidomide-Dexamethasone regimen restarted in a dose of 250 mg /day and 40mg for 4 days a week, respectively, and the response was monitored using the parameters described previously.

After 10 weeks of therapy, Bence-Jones proteinuria was absent, serum calcium was 9.8 mg/dl, plasma cells decreased in bone marrow aspirate, no new lesion was detected on radiological examination, no worsening of clinical signs and symptoms was seen and no considerable relief in pain was gained. The patient was on the same regimen and had monthly routine health checkups within the past 6 months.

## Discussion

 Multiple myeloma is characterized by malignant clone of plasma cells invading bone marrow. The patient had a marrow plasmacytosis of 40%, serum IgG component of 5.09 gm/dl, Bence-Jones proteinuria along with multiple lytic bone lesions on X-ray, thereby satisfying two major criteria and one minor criterion for the diagnosis of Multiple Myeloma^6^. Based on these criteria in a clinical setting of symptomatic disease, the patient was diagnosed with multiple myeloma.

Previous reports have documented cases of multiple myeloma during pregnancy, one such report documenting a live child birth. The treatment of multiple myeloma during pregnancy has always been a dilemma, and the role of newer agents like interferon, thalidomide and lenalidomide warrants termination of pregnancy. Thalidomide guidelines clearly indicate that it should not be taken during pregnancy and there must be at least two contraceptive methods^5^. The failure rate for termination of pregnancy using vacuum evacuation is less than 1%, and complications like incomplete evacuation, perforation and excessive bleeding have rarely been reported^7^. Unfortunately, this complication happened in our case and she ultimately underwent hysterectomy.

The role of dexamethasone- thalidomide regimen in inducing remission in newly diagnosed multiple myeloma patients have been reported to be 63% by Rajkumar et al.^8^ .Similarly, advanced and refractory myeloma has shown better results with thalidomide^2^^,^^9^**.**

Our patient had advanced myeloma and though the drug was discontinued due to retained products of conception, restarting the drug after two months of hysterectomy ensured a significant improvement. 

The hormones estrogen and progesterone are normally present in a woman, albeit in varying proportions, but β-hCG is linked to pregnancy and gestational trophoblastic diseases. The role of β-hCG as a detrimental factor in achieving a full and effective thalidomide-dexamethasone treatment needs to be evaluated with further studies. 

## CONCLUSION

 Thalidomide-based treatment for multiple myeloma in a female, who is not desirous of pregnancy, warrants an absolute method of contraception.

Thalidomide- Dexamethasone combination can be restarted even after an initial discontinuation and an effective response can occur.

The role of β-hCG, a detrimental factor to thalidomide dexamethassone treatment, needs to be evaluated.
